# Acknowledgement to Reviewers of *Veterinary Sciences* in 2019

**DOI:** 10.3390/vetsci7010011

**Published:** 2020-01-20

**Authors:** Veterinary Sciences Editorial Office

**Affiliations:** MDPI, St. Alban-Anlage 66, 4052 Basel, Switzerland

The editorial team greatly appreciates the reviewers who have dedicated their considerable time and expertise to the journal’s rigorous editorial process over the past 12 months, regardless of whether the papers are finally published or not. In 2019, a total of 106 papers were published in the journal, with a median time to first decision of 30 days and a median time from submission to publication of 51 days. The editors would like to express their sincere gratitude to the following reviewers for their generous contribution in 2019:
Abidu-Figueiredo, MarceloLopes, Ana Patrícia AntunesAchenbach, Jenna E.López-Gatius, FernandoAhlstrom, ChristinaLujan, LluisAmos, WilliamLunney, Joan K.Antipova, VeronicaLutta, Harrison OsundwaAparacio, Pedro MayorMaglio, MelaniaAranaz, AliciaMahony, Timothy JohnArechiga-Ceballos, NidiaMantzoukas, SpiridonAwosile, BabafelaMao, DaganAzaizeh, HassanMaranesi, MargheritaAzam, Aa HaerumanMarc, DanielBarlow, JohnMarconato, LauraBartlett, PaulMarletta, DonataBax, CarmenMartano, MarinaBazzano, MarilenaMartens, AnnBellini, SilviaMartínez-Sánchez, NoeliaBertazzolo, WalterMartini, Filippo MariaBertelloni, FabrizioMassari, FedericoBeyhan, SinemMaterniak-Kornas, MagdalenaBezos Garrido, JavierMathews, Karol AnnBochniarz, MariolaMatumba, LimbikaniBøgwald, JarlMaurelli, Maria PaolaBongiovanni, LauraMcCleary-Wheeler, Angela L.Borzacchiello, GiuseppeMcdonald, JenniderBovera, FulviaMcKinley, Trevelyan J.Bown, KevinMcMurray, David N.Breeze, BethMehlhorn, HeinzBritti, DomenicoMelendez, PedroBrown, DavidMeling, Daryl D.Bueno-Cavanillas, AuroraMerino, Eva Maria PerezBulatov, EmilMeyer-Aurich, AndreasBulysheva, Anna A.Mila, HannaByrne, AndrewMokhtaria, KouidriCai, DeminMoldal, Elena RegineCambier, CaroleMoreau, MaximCameron, DanielMoutelíková, RomanaCannon, Claire M.Mullin, ChristineCarrillo, JenifferNeira, Victor M. N.Cassidy, JosephNguyen, FrédériqueCastiglioni, BiancaNichols, MeginCerecetto, HugoNiedringhaus, Kevin D.Chen, Yi-NingNuttall, Patricia A.Chenais, ErikaOblak, MichelleCheng, KeOkumura, HirokiChochlakis, DimosthenisOzawa, MakotoClarke, JonathanPaes, GeertConnor, MelaniePaixão, Tatiane AlvesCortese, LauraParambeth, Joseph CyrusCortey, MartiPavicich, Maria AgustinaCrasta, ManuelaPelizzo, GloriaCribb, Alastair E.Penrith, Mary-LouiseCutrignelli, Monica IsabellaPereira, Maria De LourdesCwynar, PrzemyslawPerrucci, StefaniaDamiaans, BertPerry, Michael J.Dance, David A. B.Peterson, Karianne LievaartDandrieux, Julien R. S.Phalen, DavidDark, MichaelPlaza-Díaz, JulioDatan, EmmanuelPozzo, LuisaDavail, BlandineProsheva, ValentinaDe Biase, DavideProvot, ThomasDe Briyne, NancyQiu, Hua-JiDe Rooster, HildeRaftery, Alexandra G.Del Chicca, FrancescaRampazzo, AntonellaDevriendt, NausikaaRanadheera, SenekaDewell, GrantRavera, IvanDias-Pereira, PatriciaReavill, DruryDkhar, Hedwin KitdorlangReed, Kevin F. M.Domínguez Rebolledo, Álvaro EfrénReif, Kathryn E.Dooley, Laura M.Reifinger, MartinDorman, David C.Ressel, LorenzoDouet, Jean-YvesRiad, BouzidDrigo, MicheleRiva, FedericaDriver, AshleyRobertson, IanDumetre, AurélienRönnberg, HenrikDuran, Sue HudsonRose, NicolasDuszka, KalinaRoura, XavierDvorak, CherylRudd, PennyEda, ShigetoshiSacco, JamesEichwald, CatherineSakkas, HerculesElmi, AlbertoSamy, AhmedEl-Sheikh Ali, HossamSanad, YasserEmam, Mahmoud AbdelghaffarSandro, RolesuFan, JianglinSani, Marc-AntoineFasina, Folorunso OludayoŠarkan, BojanFei, Andrew Chang-YoungSaxena, VikasFerreira, FernandoSchaefer, MichaelFischer, AmySchildgen, OlivierFonseca-Alves, Carlos EduardoSchmidt-Bleek, KatharinaFrossard, Jean-PierreSchoiswohl, JuliaFthenakis, GeorgeSchotthoefer, Anna M.Gantenbein, BenjaminSchwarz, TobiasGiacomo, GnudiScribante, AndreaGiger, UrsSecott, TimGiovanardi, DavideSeyfang, JemmaGómez-Arrue, JavierShil, NirajGoodla, LavanyaShimada, AkinoriGordon, JessicaShiratori, HidetakaGordon, Stephen V.Simulundu, EdgarGoyal, RajniSingh, PallaviGoy-Thollot, IsabelleSinha, AmitGoździewska-Harłajczuk, KarolinaSiniscalco, DarioGrant, IreneSlana, IvaGunesekera, AmilaSmith, DaleHafez, Hafez MohamedSmith, EdHalos, LenaigSnyder, Orman (Larry)Ham, KathleenSopena, JoaquínHaney, EvanSpecht, AndrewHariharan, HarryStent, AndrewHasbun, JaimeSuárez, Cinta PrietoHe, ChendSuarez-Vega, AroaHe, QigaiSultan, KhaledHearn, Michael J.Sun, KunfengHeller, MartinSwelum, AymanHersperger, Adam R.Szóstek-Mioduchowska, AnnaHertkorn, Kathleen P.Szulczyński, BartoszHeuer, CordTajima, AtsushiHildreth, BlakeTakeshima, ShinnosukeHildreth, Michael B.Takizawa, NaokiHine, BradTashima, ToshihikoHofmeister, ErikTedesco, IdoloHotea, IonelaThaler, RobertHubková, BeátaThapa, SantoshHuneau, AdelineThompson, NathanaelIlyinskii, Petr O.Tibor, MagyarInga, AlbertoToledo, AlvaroIorio, Ronald M.Tomaszewska, EwaIsoda, NorikazuToomer, Gabriela Chavez-CalvilloJahns, HanneTothova, CsillaJaja, IshmaelTsairidou, SmaragdaJansen, Christine A.Tufarelli, VincenzoJansson, DésiréeVentrella, DomenicoJefferson, Wendy N.Verguts, JasperJensen, KirstyVezzosi, TommasoJeyanathan, JeyamalarVillari, SaraJung, Dong-InVrentas, Catherine E.Kajamies, AnuWales, AndrewKang, SeongWatkins, CraigKawaji, SatokoWeiss, Mark L.Kennedy, Richard B.Wendelburg, KristinKeuling, OliverWhelan, StephenKilcawley, KieranWhite, Nathaniel A.Kitajima, ShujiWhittington, Richard J.Klotz, JamesWojtyczka, RobertKnauer, WhitneyWolf, PeterKoralur, MunegowdaWooton-Kee, Clavia RuthKrockenberger, MarkWu, Jack H. Z.Kumar, AnandWu, Meng-YuKupke, AlexandraWycislo, KathrynLaddomada, AlbertoXie, QingmeiLaham-Karam, NihayYang, Run-jun YangLahbib-Mansais, YvetteZaleski, HalinaLei, ZhihaiZani, LauraLeite, Fábio Pereira LeivasZappulli, ValentinaLiao, Albert T.Zarogoulidis, PaulLiegner, KennethZhalniarovich, YauheniLinhares Martins Gabriel, Dra. ÁureaZhang, GuanshiLipschutz-Powell, DebbyZhao, ShayingLiu, JingboZmora, PawelLo, SzechengZou, Wei

